# Treatment of immunothrombosis dysregulation: high-dose corticosteroids is not the good option

**DOI:** 10.1186/s13613-023-01158-1

**Published:** 2023-06-28

**Authors:** Julie Helms, Julien Poissy, Pierre-François Dequin, Jean-François Timsit

**Affiliations:** 1 Université de Strasbourg (UNISTRA), Faculté de Médecine, Strasbourg, France; 2 Hôpitaux Universitaires de Strasbourg, Service de Médecine Intensive-Réanimation, Nouvel Hôpital Civil, 1, Place de L’Hôpital, 67091 Strasbourg Cedex, France; 3 Université de Lille, Inserm U1285, CHU Lille, Pôle de Médecine Intensive-Réanimation, CNRS, UMR 8576—UGSF—Unité de Glycobiologie Structurale Et Fonctionnelle, 59000 Lille, France; 4 INSERM U1100, Université de Tours, Tours, France; 5 Service de Médecine Intensive-Réanimation Et INSERM CIC 1415, CHU de Tours, Tours, France; 6 Assistance Publique Hôpitaux de Paris (AP-HP), Bichat Hospital, Medical and Infectious Diseases ICU (MI2), 75018 Paris, France; 7 IAME—U1137, University Paris-Cité, INSERM, 75018 Paris, France

Dear Editor,

We read with great interest the article of Jonmarker et al. [1].

Patients with severe COVID-19 were early identified as being at higher risk of developing thrombotic complications than other critically ill patients. Consequently, clinical trials have attempted to increase the anticoagulant regimen to intermediate or therapeutic doses or to test alternative anticoagulant and antithrombotic agents. Unfortunately, as with sepsis-induced coagulopathy (SIC), clinical trials testing anticoagulants are disappointing, with inconclusive or negative results.

Several issues should be raised to understand why, despite a relevant problem, the proposed solution may not have been the right one, in both SIC and COVID-19.

In sepsis, most of the trials suffered from the same flaws: *first*, the trials have often included septic patients whether or not they have SIC. However, it does not make sense to anticoagulate a patient who does not have excessive coagulation activation and disseminated microthrombi (Fig. 1). Patients should therefore be stratified and included in a trial only if they are likely to benefit, i.e., only if they have a positive SIC score. *Second,* potential anticoagulant treatment should be tailored to the stage of coagulation activation. In bacterial sepsis, the host response to pathogen invasion involves close interactions between innate immunity and coagulation—termed immunothrombosis—that allow recognition, containment, and destruction of pathogens. These defense mechanisms can however be dysregulated, leading to excessive coagulation activation with defective fibrinolysis, resulting in disseminated intravascular coagulation [2]. In a very early stage of adaptive hemostasis, anticoagulation of septic patients—and thus prevention of immunothrombosis—may be detrimental, whereas in a later thrombotic stage, it may become beneficial. In addition to appropriate patient stratification, it is therefore necessary to identify the correct therapeutic window for anticoagulant treatment.

**Fig. 1:**
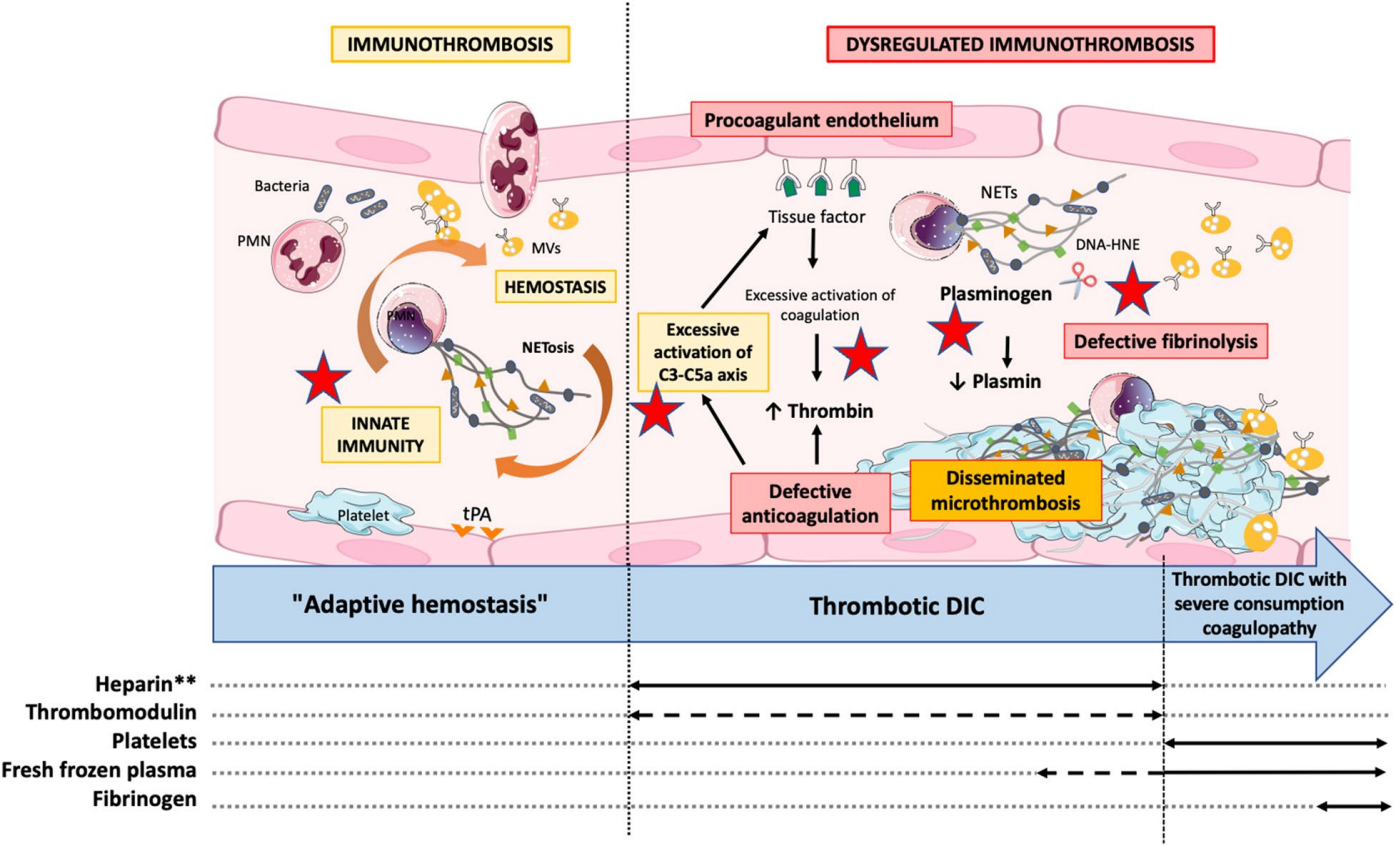
Immunothrombosis refers to the interactions between innate immunity and hemostasis, and contributes to host defense against the pathogen. Sepsis-induced coagulopathy results from immunothrombosis dysregulation, leading to an excessive generation of thrombin and disseminated microthrombi. Treatment of sepsis-induced coagulopathy should depend on coagulation activation stage, although none of the presented treatment has been validated yet. New therapeutic targets (red stars) are currently being investigated and might lead to improve the management of SIC in the next years. *HNE* human neutrophil elastase, *PMN* polymorphonuclear neutrophil, *MVs* microvesicles, *NETs* neutrophil extracellular traps, *tPA* tissue plasminogen activator

This interplay between excessive innate immunity activation, involving C3–C5 axis activation, and thrombosis has also been shown in viral sepsis, especially in COVID-19 [3]. But history has repeated itself, in a context of great difficulty of conducting trials of high methodological quality during a pandemic period. *First*, some trials have included patients with COVID-19 of varying severity, corresponding to different immunothrombosis and respiratory phenotypes. *Second*, the rapid evolution of the pandemic, the demonstration of efficacy of therapeutics that potentially interact with hemostasis (corticosteroids, immunomodulators), the improvement of supportive therapies, and the evolution of virus strains have made it difficult to compare trials conducted during different waves. *Third*, the practice of enhanced anticoagulation has spread rapidly, in line with several available recommendations despite their low-level of evidence.

The interaction between immunomodulation and a reduction in the risk of immunothrombosis seems theoretically relevant, but is not evident in bedside clinical practice. Some data regarding the use of anti-IL6 and a possible increased risk of procoagulant profile call for caution. Anti-JAK molecules are also known to increase thrombotic risk in long-term treated patients, and this has led to the exclusion of patients with documented thromboembolism on admission from the trials testing these molecules in COVID-19. By contrast, in a retrospective, observational before/after bi-centric cohort study among ICU patients hospitalized for severe COVID-19 and receiving therapeutic anticoagulation by unfractionated heparin, dexamethasone (DXM) was associated with a decrease in both proinflammatory and procoagulant profile. Following the hypothesis of a dose–effect of DXM, Jonmarker et al*.* [1] conducted a post hoc analysis of the blinded randomized COVID STEROID-2 trial comparing the efficacy of 12 mg *vs.* 6 mg DXM daily and assessed a composite outcome of death or thromboembolism in patients with critical COVID-19. More than 350 patients were included, but the study failed to show any difference between the two corticosteroid doses. This result is consistent with that observed in the COVIDICUS study [4]. However, both studies were not specifically designed to explore the risk of thrombo-embolic diseases. The 8.7%-observed incidence of thrombosis is not consistent with the up to 20% incidence observed in studies specifically designed to examine the risk of thrombosis in SARS-CoV-2 acute respiratory failure [5]. The potential impact of high-dose DXM on immunothrombosis is also confounded by the lack of systematic screening for thrombo-embolic events prior to randomization. In addition, the strategy of anticoagulant prophylaxis, which may affect venous thrombo-embolic risk, was not homogeneous and may affect the effect of high-dose DXM.

Despite these shortcomings, the negative result of this secondary analysis of COVID STEROID-2 argues for considering all potential targets of immunothrombosis in the future. If immunomodulatory treatments have an effect on thrombotic events, it can only be shown by a prospective study standardizing the associated treatments and systematically looking for venous thrombosis. As for anticoagulant treatment, we have to keep in mind that a curative treatment cannot decrease thrombotic events without increasing bleeding events. It is therefore a benefit/risk assessment that must be conducted, taking into account confounding factors and, as mentioned, stratifying patients according to precise diagnostic and severity criteria.

## Data Availability

Not applicable.

## Abbreviations

DXM: Dexamethasone
HNE: Human neutrophil elastase
PMN: Polymorphonuclear neutrophil
MVs: Microvesicles
NETs: Neutrophil extracellular traps
SIC: Sepsis-induced coagulopathy
tPA: Tissue plasminogen activator
